# Beneficial Microorganisms in Sustainable Agriculture: Harnessing Microbes’ Potential to Help Feed the World

**DOI:** 10.3390/plants11030372

**Published:** 2022-01-29

**Authors:** Amelia C. Montoya-Martínez, Fannie Isela Parra-Cota, Sergio de los Santos-Villalobos

**Affiliations:** 1 Laboratorio de Biotecnología del Recurso Microbiano, Departamento de Ciencias Agronómicas y Veterinarias, Instituto Tecnológico de Sonora (ITSON), 5 de febrero 818 Sur, Col. Centro, Ciudad Obregón 85000, Mexico; cristina_montoya14@hotmail.com; 2 Campo Experimental Norman E. Borlaug, Instituto Nacional de Investigaciones Forestales, Agrícolas y Pecuarias (INIFAP), Norman E. Borlaug Km. 12, Ciudad Obregón 85000, Mexico

The global population is projected to increase to near 10 billion people by the year 2050 [1,2]; therefore, food demand will increase. Different projections show that feeding that world population would require raising overall food production by 25 to 70 percent between now and 2050 [2,3]—some authors go as far as 100% [4]. In order to produce that amount of food and other non-food agricultural goods, intensified and extended agricultural production is needed. These practices have led to many environmental problems, such as the degradation of natural resources, such as soil and water, the loss of microbiome diversity, the proliferation of new phytopathogens, pests, and weeds, and lower agro-ecosystem fertility, and is contributing to climate change [4,5,6]. Increasing agricultural production in ways that do not compromise environmental integrity and/or human health continues to be a big challenge to date.

The sustainable intensification of agricultural production is needed to meet food demand while maintaining functioning and healthy ecosystems. Currently, biotechnological tools are being used to complement conventional crop management; among them, the use of bio-products containing beneficial microorganisms, also known as microbial inoculants or bioinoculants, is gaining importance in this matter [7]. Fortune Business Insights has published a report stating that the global market size for agricultural bioinoculants was USD 4.27 billion in 2019 and is projected to reach 11.81 billion by 2027, with an annual growth rate of 14.27% [8]. Bioinoculants are eco-friendly and sustainable bio-products containing beneficial microorganisms that, when applied to seeds, plant surfaces, or soil, have the capability of promoting plant growth by increasing the supply or availability of nutrients to the host plant or by protecting them from biotic and/or abiotic stresses [7,9,10]. Beneficial microorganisms used in bioinoculants can be grouped as plant-growth-promoting microorganisms (PGPMs) or biological control agents (BCAs) [9,11,12]. According to their mechanisms of action, they can also be cataloged as nitrogen fixers, phosphate solubilizers, phytostimulators, inducers of plant resistance, and antagonists of phytopathogens, among other groups (Figure 1) [11,13].

The integration of beneficial microorganism–plant interactions into crop production programs represents a promising sustainable solution to improve agricultural production. However, the success of bioinoculants applied in the field is highly determined by the scientific research and agro-biotechnological innovations behind them. Therefore, the study of and research focused on understanding the complex interactions between beneficial microorganisms, plants, and their environment, as well as the bioprospecting needed to massively exploit these interactions, are crucial to moving knowledge from labs to fields as commercial effective products.

As its Guest Editors, we are confident that this Special Issue will collect and inspire high-impact research activities, offering new insights into wide-ranging topics and approaches to the role of often-unexploited microbiota in agriculture for providing sustainable alternatives to warrant global food security.

## Figures and Tables

**Figure 1:**
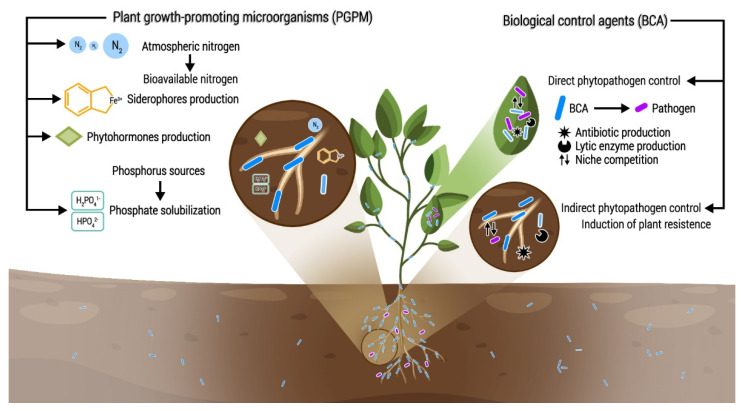
Examples of action mechanisms by beneficial microorganisms in interactions with plants.
